# Visible-light-driven Photocatalytic N-arylation of Imidazole Derivatives and Arylboronic Acids on Cu/graphene catalyst

**DOI:** 10.1038/srep12005

**Published:** 2015-07-20

**Authors:** Yan-Li Cui, Xiao-Ning Guo, Ying-Yong Wang, Xiang-Yun Guo

**Affiliations:** 1 State Key Laboratory of Coal Conversion, Institute of Coal Chemistry, Chinese Academy of Sciences, Taiyuan 030001, China; 2 University of the Chinese Academy of Sciences Beijing 100039, China

## Abstract

N-aryl imidazoles play an important role as structural and functional units in many natural products and biologically active compounds. Herein, we report a photocatalytic route for the C-N cross-coupling reactions over a Cu/graphene catalyst, which can effectively catalyze N-arylation of imidazole and phenylboronic acid, and achieve a turnover frequency of 25.4 h^−1^ at 25 ^o^C and the irradiation of visible light. The enhanced catalytic activity of the Cu/graphene under the light irradiation results from the localized surface plasmon resonance of copper nanoparticles. The Cu/graphene photocatalyst has a general applicability for photocatalytic C-N, C-O and C-S cross-coupling of arylboronic acids with imidazoles, phenols and thiophenols. This study provides a green photocatalytic route for the production of N-aryl imidazoles.

The C-N cross-coupling has been recognized as one of the most important transformations in organic chemistry because it can be conveniently used to synthesize a wide range of organics including many natural products and pharmaceuticals. N-aryl imidazoles play an important role as structural and functional units in many natural products and biologically active compounds^1^,^2^,^3^,^4^,^5^. As an efficient method to produce N-aryl imidazoles, copper-mediated C-N bond formation via the cross-coupling of arylboronic acids and imidazoles using stoichiometric Cu(OAc)_2_ and pyridine has become an important synthetic strategy since the initial reports by Chan and Lam groups^6^,^7^,^8^. Later, Collman and co-workers reported that Cu(II) complexes with nitrogen-chelating bidentate ligands could catalyze the coupling of imidazoles without the addition of any base at room temperature^9^. Recently, simple copper salts were also found to promote the coupling of arylboronic acids with imidazoles in protonic solvents^10^. Although these homogeneous processes using soluble Cu complexes as the catalysts exhibit high catalytic efficiency, the difficulty in separating the catalysts from the reaction mixtures is still a serious problem. Comparing with the homogeneous processes, the routes employing recyclable heterogeneous catalysts would be efficient and environmentally friendly^11^,^12^. For example, Kantam *et al.* found that N-arylation of imidazoles and arylboronic acids was accomplished with copper-exchanged fluorapatite in methanol^12^. However, the activity of heterogeneous catalysts still remains to be improved.

The localized surface plasmon resonance (LSPR) effect is a collective oscillation of conduction electrons in metallic nanoparticles, which resonate with the electromagnetic field ofincident light in visible light range^13^,^14^,^15^. The conduction electrons of the nanoparticles of gold (Au), silver (Ag) and copper (Cu) can gain visible light energy through the LSPR effect to produce energetic “hot” electrons, which facilitate the reaction process and improve the yield of chemical synthesis under mild conditions^16^. Studies on light-driven reactions catalyzed by plasmonic metal nanoparticles have formed the basis of a new and fast-expanding field in green photocatalysis^17^,^18^,^19^,^20^,^21^. Linic’s group found that plasmonic nanostructures of silver can concurrently use low-intensity visible light and thermal energy to drive catalytic oxidation reactions-such as ethylene epoxidation, CO oxidation, and NH_3_ oxidation at lower temperatures^19^. Sarina *et al.* reported that the Au-Pd alloy nanoparticles can strongly absorb light due to the LSPR effect of Au and efficiently enhance the conversion of some reactions such as Suzuki-Miyaura cross coupling^20^. Huang *et al.* considered that activation of oxygen on Au and Ag Nanoparticles can be assisted by LSPR in oxidation reactions since the effective injection of excited energetic electrons into oxygen molecules to form strongly adsorbed oxygen molecule anions^21^. Previously, we found that copper nanoparticles supported on graphene can controllably catalyze the coupling reactions of nitroaromatics to corresponding azoxy or azo compounds under visible light irradiation^22^. Herein we report that Cu/graphene catalyst exhibits excellent photocatalytic activity for N-arylation of imidazoles and arylboronic acids at room temperature under visible light irradiation.

## Results and Discussion

Graphene supported copper nanoparticles were prepared by reducing Cu_2_O/graphene composites^22^. The N-arylation of imidazole and phenylboronic acid was conducted in methanol at room temperature in the atmosphere of O_2_. A 300W Xe lamp (wavelength from 400–800 nm, the light spectrum is shown in Figure S1) was employed as light source to irradiate the reaction system containing 50 mg Cu/graphene catalyst (Figure S2). In the Cu/graphene catalyst with a Cu loading of 5 wt%, Cu nanoparticles were uniformly dispersed on the graphene sheets, and had a mean diameter of 15 nm (Figure S3A and B). However, the mean size varies with the Cu loading on graphene. It is ~7 nm for 3wt% Cu/graphene, and ~52 nm for 7 wt% Cu/graphene (Figures S3C and D). The patterns of X-ray Photoelectron Spectroscopy (XPS) are similar for the Cu/graphene catalysts with different Cu loadings. The binding energies of Cu 2p_1/2_ at around 952.2 eV and Cu 2p_3/2_ at 932.6 eV, respectively, can be attributed to the Cu^0^ state (Figure S4), confirming that Cu exists on the graphene sheets as the metallic phase. X-ray diffraction patterns (Figure S5) can further prove this. In the UV-vis spectra (Figure S6), the absorption peak at approximately 560 nm is due to the LSPR absorption of Cu nanoparticles. Meanwhile, the absorption intensity of Cu/graphene increases with the Cu loading increasing. The above results suggest that copper in the Cu/graphene catalyst is in the metallic state rather than oxides. Graphene is a two-dimensional network of sp^2^-bonded carbon atoms^23^,^24^, and the delocalized electrons in graphene can move freely in the network with a low resistance^25^. Generally, Cu nanoparticles are easily oxidized to Cu_2_O or CuO in air or in the presence of trace molecular oxygen. However, they can become stable when Cu nanoparticles are supported on graphene sheets^22^. The stabilization possibly originates from the interaction of electrons in Cu nanoparticles and in graphene. Additionally, the carbon vacancies or dangling bonds in graphene can influence the electronic structure of Cu on graphene to improve their chemical stability and keep them existing as metallic phase.

**Figure 1:**
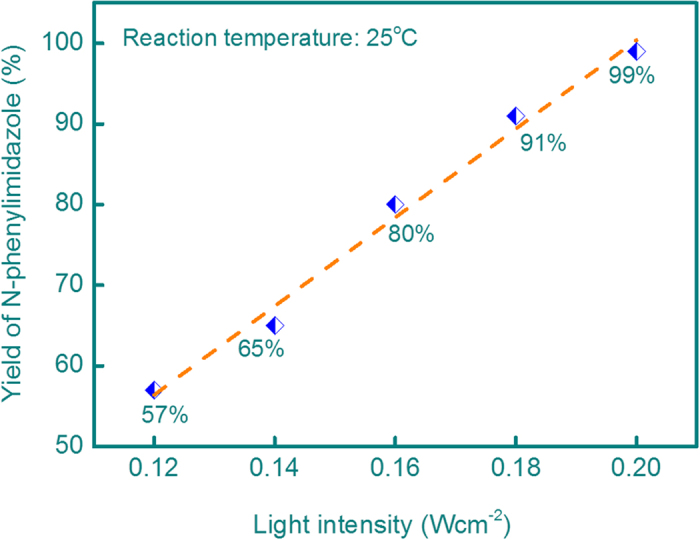
Dependence of the catalytic activity of 5 wt% Cu/graphene for the N-arylation of imidazole and phenylboronic acid on the irradiation intensity .

For the N-arylation of imidazole and phenylboronic acid, the Cu/graphene catalyst exhibited excellent photocatalytic activity under the given conditions ([a] in Table 1). The yield of N-phenylimidazole is 99% and the turnover frequency (TOF) is 25.4 h^−1^. Control experiment reveals that the Cu/graphene catalyst in dark can only achieve a trace yield of N-phenylimidazole (<0.1%), suggesting that the high activity of the Cu/graphene for the coupling reaction results from the light irradiation. No reaction product is detected using only graphene as the photocatalyst, and it is not detected too in the absence of any catalysts (whether Cu/graphene or pure graphene). The reaction did not occur when employing Cu^2+^ (copper acetate) and graphene with equal molar ratio as catalyst under irradiation. The above results suggest that the active phase for this reaction is metallic Cu nanoparticles. Cu/SiO_2_ and Cu/TiO_2_ catalysts with the same Cu loading as Cu/graphene were also prepared under the protection of Ar. However, the yield of N-phenylimidazole over these two catalysts is only 16% and 29%, respectively. This is mainly resulted form that metallic Cu active phase on SiO_2_ or TiO_2_ surface was easily oxidized into Cu_2_O and CuO in the presence of O_2_ in the reaction system. When the light intensity was reduced from 0.2 Wcm^−2^ to 0.18 Wcm^−2^, 0.16 Wcm^−2^, 0.14 Wcm^−2^ and 0.12 Wcm^−2^ but all the other experimental conditions remained unchanged, the yield of N-phenylimidazole linearly decreased from 99% to 91%, 80%, 65% and 57%, respectively (Fig. 1). It is thus obvious that the reaction was driven by the light irradiation.

The photocatalytic activity of 5 wt% Cu/graphene is superior to 3 wt% and 7 wt% Cu/graphene catalysts in the N-arylation of imidazole and phenylboronic acid. The yield of N-phenylimidazole on 3 wt% and 7 wt% catalysts is 82% and 87%, respectively. Catalysts of 5 wt% and 7 wt% Cu/graphene exhibit similar LSPR absorption but the Cu particles in the 7 wt% catalyst are much larger than those in the 5 wt% catalyst. The larger Cu particles have smaller specific surface area and thus less active sites where the catalytic reaction takes place. The Cu particles in 3wt% Cu/graphene are smaller in size but show obviously weaker LSPR absorption comparing with those of the other two catalysts (Figure S6). The weaker light absorption results in a lower activity since the catalytic process is mainly driven by light.

**Figure 2:**
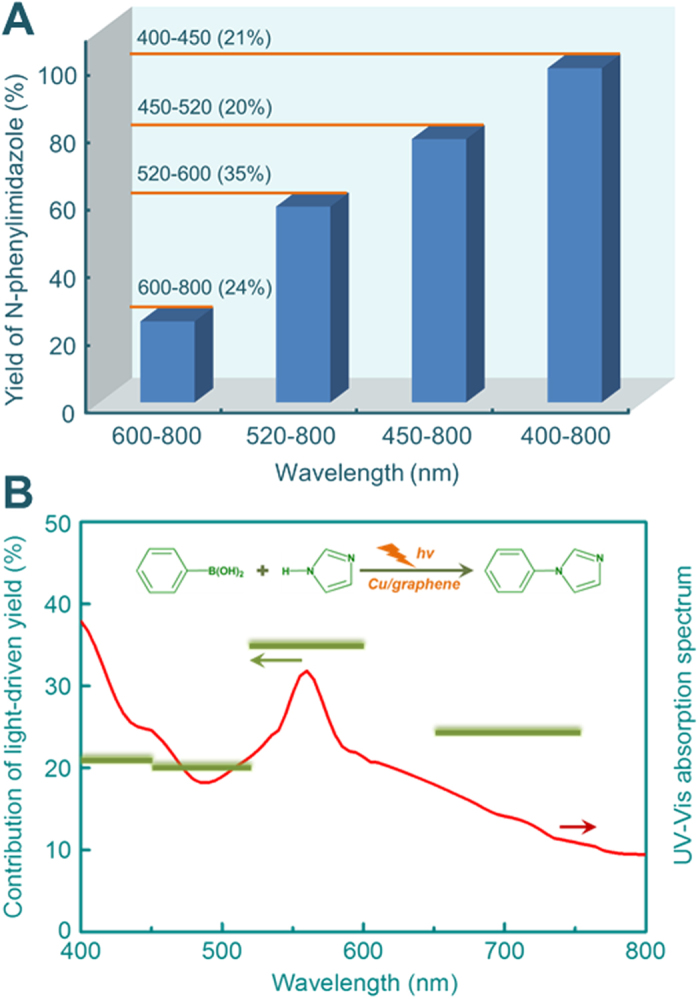
Dependence of N-phenylimidazole yield on the irradiation wavelength (**A**), and the action spectrum of the photocatalytic reaction, in which the light driven conversion is plotted against the irradiation wavelength (**B**).

The dependence of the catalytic activity on the irradiation wavelength was illustrated in Fig. 2. A series of optical low-pass filters were employed to block light below a specific cut-off wavelength. For example, the 450 nm optical filter blocks the wavelength below 450 nm and over 800 nm, in other words, the light irradiating the reactor has a wavelength range from 450 to 800 nm. Without any filters, the irradiation of the light with wavelengths ranging from 400 to 800 nm gives a N-phenylimidazole yield of 99%. The yield decreases to 78%, 58% and 24% when the wavelength range of the irradiation is 450–800, 520–800, and 600–800 nm, respectively. Since the yield of N-phenylimidazole in the dark is negligible, the contribution of 400–450 nm light accounts for about 21% ((99–78)/99 × 100%) in the total light-induced yield. Similarly, the light in the wavelength range of 450–520, 520–600 and 600–800 nm, respectively accounts for 20%, 35% and 24% of the light-induced yield (Fig. 2A). These values agree well to the UV-visible absorption spectrum of the Cu/graphene catalyst (Fig. 2B). Because the Cu/graphene catalyst has a strong absorption at about 560 nm, the light in the wavelength range of 520–600 nm contributions the highest light-induced conversion. This further confirms that the light absorbed by Cu nanoparticles is the major driving force of the reaction.

**Figure 3:**
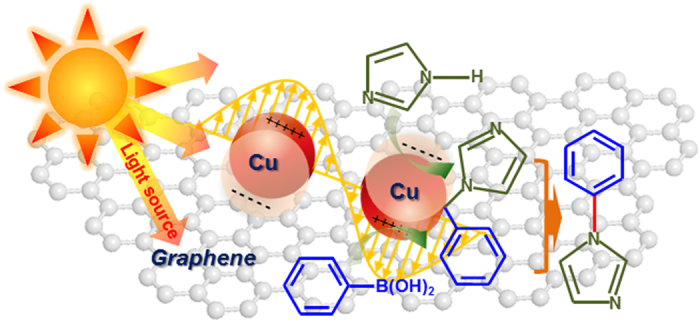
Schematic mechanism of the N-arylation reaction of imidazole and phenylboronic acid.

5,5-Dimethyl-1-pyrroline N-oxide (DMPO) is an electron-trapping agent that can capture electrons from Cu nanoparticles^26^. When 0.5 mL of DMPO was added to the N-arylation reaction of imidazole and phenylboronic acid, no product can be detected, but some by-products are formed due to self-coupling or oxidation of phenylboronic acid. Due to the LSPR effect, the electron density in Cu particles is polarized, and there will be a charge heterogeneity at the surface of Cu particles, with both relatively electron-rich sites and slightly positively charged sites present^27^,^28^. The former can easily adsorb electrophilic imidazole molecules. The conduction electrons of Cu nanoparticles gain the irradiation energy and become high energy electrons, and the energetic electrons can inject into adsorbed imidazole molecules, facilitating the cleavage of N-H bonds to form Cu-N(imidazole) complexes^29^,^30^. Meanwhile, the latter can assist to cleave the C-B bonds in phenylboronic acid molecule to form Cu-Ar complexes^31^,^32^. Then the redox-activated species couple to finish the C-N crossing coupling. A schematic mechanism on the N-arylation reaction of imidazole and phenylboronic acid is illustrated in Fig. 3. Moreover, graphene can also absorb light (Figure S6). Jarillo-Herrero *et al.* found that hot carrier-assisted intrinsic photo response in graphene can generate strong photocurrent^33^. The work function (WF) of graphene is about 4.5 eV, and the WF of Cu is 4.65 eV. Due to the different WFs, a built-in potential of 0.15 eV is formed near the junction between graphene and Cu. Because the WF of graphene is lower than Cu, the hot-electrons with high energy can easily transfer to Cu from graphene^34^,^35^,^36^,^37^. This can also result in a collection of energetic electrons at the Cu sites on the nanoparticle surface to further accelerate the reaction.

Elevated reaction temperature also accelerates the catalytic activity, which is an important feature of the photocatalytic process on plasmonic metal nanoparticle catalysts^38^. We respectively conducted the reaction at 80 ^o^C and 120 ^o^C under a constant light intensity of 0.2 Wcm^−2^. The reaction can be completed in 25 and 10 min, respectively. Both yields of N-phenylimidazole are 99%, and the TOF is 61 h^−1^ and 152 h^−1^, respectively. The yield is only 21% and 47% without irradiation at 80 ^o^C and 120 ^o^C, respectively, suggesting that light-driven yield can contribute the most of total yield even at high temperture. However, elevated temperature can cause a redistribution of the conduction electrons of Cu nanoparticles into higher energy levels^39^,^40^. Those electrons at higher levels can gain additional energy through light absorption when irradiated. This increase in energy further enhances the probability that the energetic electrons activate the reactant molecules adsorbed on the surface of nanoparticles. Moreover, the relative population of reactant molecules in excited states increases with temperature rising according to the Bose-Einstein distribution^20^. This means that the reactant molecules will require less energy to overcome the activation barrier and this energy could be easily provided by light irradiation. These results indicate that the electrons of Cu nanoparticles can effectively couple thermal and photonic energies to drive the chemical reactions.

To test the applicability of Cu/graphene photocatalyst, we employed various arylboronic acids to react with different imidazole derivatives. The results are summarized in Table 1. The presence of electron-donating groups in imidazole or benzimidazole almost did not affect the reaction yield (entries 2–3). However, the electron-withdrawing groups in imidazole can greatly decrease the reaction yield (entries 4–5). The influences of electron-donating (entries 6–8) and electron-withdrawing groups (entries 9–11) in arylboronic acids are similar to the imidazole derivatives. C-O and C-S cross-coupling reactions of phenylboronic acid with phenols and thiophenols to form corresponding ethers and thioethers compounds were also investigated (Table 2). The Cu/graphene catalyst also exhibited good photocatalytic activity for these reactions. The yields of diphenyl ether and diphenyl sulfide can reach to 87% and 93%, respectively, under irradiation and the given conditions (entries 1 and 4); while they are 29% and 41%, respectively, in the dark reaction (without light irradiation). Because phenols and thiophenols often act as nucleophiles, they are more inclined to adsorb on the positively charged sites of Cu nanoparticle. Therefore, the energetic electrons cannot directly interact with the adsorbed reactant molecules, and thus the reaction processes are prolonged to 10 h. However, this needs further investigation. The electron-donating (entries 2 and 5) and electron-withdrawing groups (entries 3 and 6) have opposite influences to the coupling reactions. These results demonstrate the general applicability of the Cu/graphene catalyst for photocatalytic C-N, C-O and C-S cross-coupling reactions of arylboronic acids with imidazoles, phenols and thiophenol.

**Figure 4:**
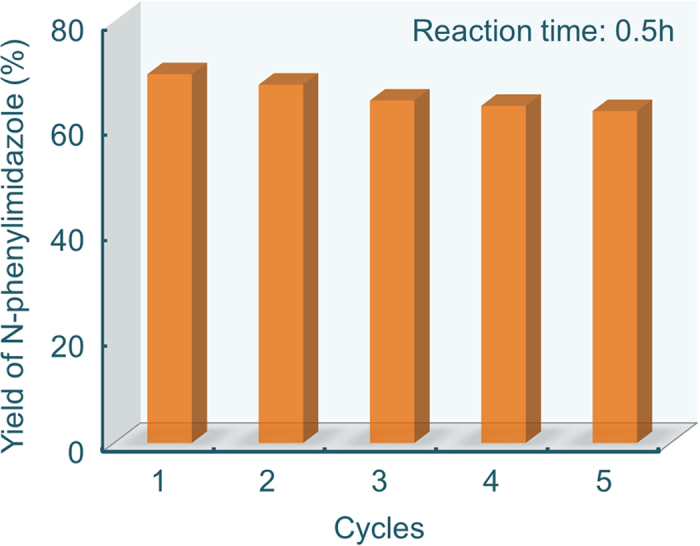
Recyclability of 5 wt% Cu/graphene catalyst in the N-arylation reaction of imidazole and phenylboronic acid.

Good recyclability is the main superiority of heterogeneous catalysts. To test the recyclability of the Cu/graphene photocatalyst in the N-arylation of imidazole and phenylboronic acid, the catalyst was reused for 5 times after filtering and drying. A slow decrease in the activity was found after five reaction cycles, from 70% at the first cycle to 62% at the 5^th^ (Fig. 4). TEM and X-ray diffraction results of the used catalyst show no obvious change in morphology or aggregation of the Cu nanoparticles and Cu phase (Figure S7). Comparing with the fresh Cu/graphene, the binding energy of Cu 2p_3/2_ (932.7 eV) in the XPS of the used Cu/graphene slightly shifts to higher value (Figure S8), which is between that of Cu^0^ (932.6 eV) and Cu^+^ (932.8 eV). Moreover, the activity of used Cu/graphene can be recovered to its initial level after it is reduced in H_2_-containing atmosphere, indicating that a slight oxidation of Cu nanoparticles occurs during the photocatalytic process. Nevertheless, the Cu/graphene shows a good recyclability in this type of photocatalytic coupling reactions.

## Conclusion

The present work demonstrates a novel photocatalytic route of the C-N cross-coupling reactions over a Cu/graphene catalyst. The Cu/graphene shows an excellent photocatalytic activity for N-arylation of imidazole and phenylboronic acid, and achieves a turnover frequency of 25.4 h^−1^ at 25 ^o^C and under the irradiation of 0.2 Wcm^−2^ visible light. The results for varieties of reaction substrates reveal the general applicability of the Cu/graphene catalyst for the C-N, C-O and C-S cross-coupling of arylboronic acids with imidazoles, phenols and thiophenols. Therefore, it provides an efficient and green photocatalytically and heterogeneously catalytic route for the production of N-aryl imidazoles.

## Methods

### Materials

All chemicals were purchased from Aladdin and were used as received.

### Preparation of 5 wt% Cu/graphene catalyst

The Cu/graphene catalyst was prepared the same as our previous work by reducing Cu_2_O/graphene composites in a mixture of H_2_ (5 vol %) and Ar at 500 ^o^C. The Cu_2_O /graphene composite were prepared via a two-step route. First, graphite oxide and copper acetate were dispersed into absolute ethanol under sonication, then the suspension was magnetically stirred to get a mixture of copper acetate and graphite oxide. In the second step, the mixture was reduced using diethylene glycol for 2 h at 180 ^o^C to obtain the Cu_2_O /graphene composite.

### Photocatalytic C-N, C-O and C-S cross-coupling reactions

The photocatalytic N-arylation of imidazole derivatives and arylboronic acids were conducted in 1 atm O_2_ atmosphere at 25 ^o^C. The reactant mixture consists of 10 mL methanol, 1 mmol imidazole derivatives, 1 mmol arylboronic acids and 50 mg 5 wt% Cu/graphene catalyst. The irradiation intensity was 0.2 Wcm^−2^ and the reaction time was 1 h unless otherwise specified. The dependence of the catalytic performance on the wavelength range of light was investigated by employing various low pass optical filters to block light below specific cut-off wavelengths while maintaining the light intensity to the reaction system unchanged. For instance, a filter with the cut-off wavelength of 450 nm can block light with wavelengths shorter than 450 nm (the system is irradiated by light with wavelengths between 450 and 800 nm). Similarly light with wavelengths in the ranges of 520–800 and 600–800 nm were applied to the reaction system using filters with cut-off wavelengths of 520 nm and 600 nm, respectively. The conditions of photocatalytic C-O and C-S cross-coupling reactions of arylboronic acids with phenols and thiophenols were same as C-N cross-coupling reactions. However, for C-O cross-coupling reactions, phenols and dichloromethane were instead of imidazoles and methanol respectively and 1.5 mmol Cs_2_CO_3_ was used; the reaction temperature is 130 ^o^C. For C-S cross-coupling reactions, thiophenols and dimethyl formamides were instead of imidazoles and methanol respectively; and the reaction temperature is 130 ^o^C.

### Recovering of the used Cu/graphene catalyst

To test the recyclability of the Cu/graphene photocatalyst in the N-arylation of imidazole and phenylboronic acid, the catalyst was reused for 5 times after filtering and drying. To better show the stability of Cu/graphene, every cyclic reaction was conducted in 0.5 h, when the reaction did not reach its equilibrium. A slow decrease in the activity was found after five reaction cycles, from 70% at the first cycle to 62% at 5th cycle. The used Cu/graphene photocatalyst after 5 times was reduced in a mixture of H_2_ (5 vol %) and Ar at 500 ^o^C for 4 h; and then it was reused in the N-arylation of imidazole and phenylboronic acid. Its activity can be recovered to its initial level.

### Characterization

The Cu/graphene catalyst was characterized by high-resolution transmission electron microscope (HRTEM, JEM-2010). The products were analyzed by BRUKER SCION SQ 456 GC-MS to measure the concentration change of reactants and products. The quantitative analysis of specific analytes was detected by SIM mode in GC-MS.

## Additional Information

**How to cite this article**: Cui, Y.-L. *et al.* Visible-light-driven Photocatalytic N-arylation of Imidazole Derivatives and Arylboronic Acids on Cu/graphene catalyst. *Sci. Rep.* **5**, 12005; doi: 10.1038/srep12005 (2015).

## Figures and Tables

**Table 1:**
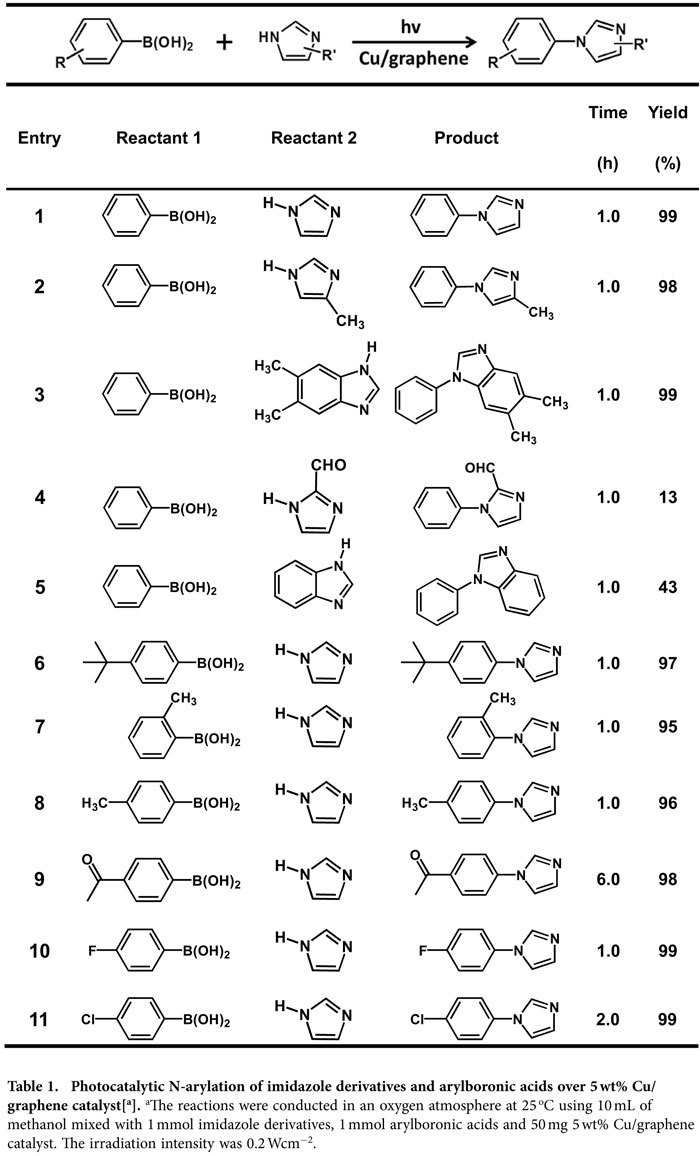
Photocatalytic N-arylation of imidazole derivatives and arylboronic acids over 5 wt% Cu/graphene catalyst[^a^].

^a^The reactions were conducted in an oxygen atmosphere at 25 °C using 10 mL of methanol mixed with 1 mmol imidazole derivatives, 1 mmol arylboronic acids and 50 mg 5 wt% Cu/graphene catalyst. The irradiation intensity was 0.2 Wcm^-2^.

**Table 2:**
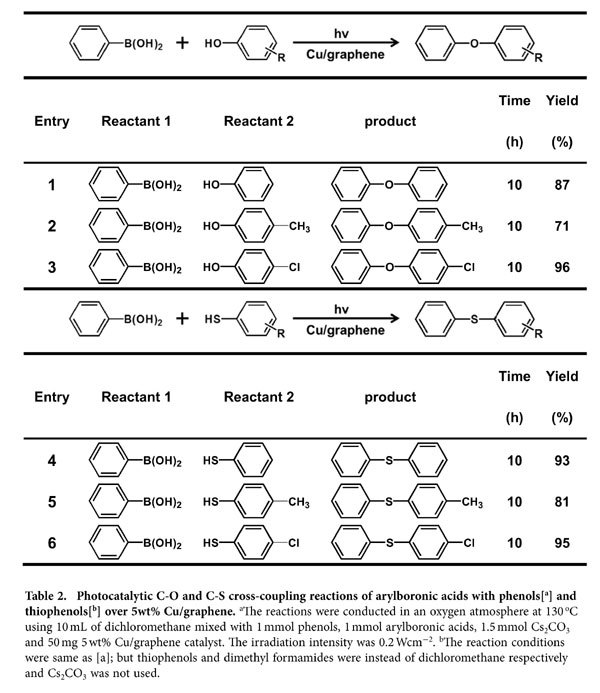
Photocatalytic C-O and C-S cross-coupling reactions of arylboronic acids with phenols[^a^] and thiophenols[^b^] over 5wt% Cu/graphene.

^a^The reactions were conducted in an oxygen atmosphere at 130 ^o^C using 10 mL of dichloromethane mixed with 1 mmol phenols, 1 mmol arylboronic acids, 1.5 mmol Cs_2_CO_3_ and 50 mg 5 wt% Cu/graphene catalyst. The irradiation intensity was 0.2 Wcm^−2^.

^b^The reaction conditions were same as [a]; but thiophenols and dimethyl formamides were instead of dichloromethane respectively and Cs_2_CO_3_ was not used.
