# Author Correction: Visible-light-driven Photocatalytic N-arylation of Imidazole Derivatives and Arylboronic Acids on Cu/graphene catalyst

**DOI:** 10.1038/s41598-024-51807-z

**Published:** 2024-01-23

**Authors:** Yan-Li Cui, Xiao-Ning Guo, Ying-Yong Wang, Xiang-Yun Guo

**Affiliations:** 1 State Key Laboratory of Coal Conversion, Institute of Coal Chemistry, Chinese Academy of Sciences, Taiyuan, 030001 China; 2 University of the Chinese Academy of Sciences, Beijing, 100039 China

Correction to: *Scientific Reports* 10.1038/srep12005, published online 20 July 2015

This Article contains errors. The Supplementary Information file, which was included with the initial submission, was omitted from the original version of this Article. The Supplementary Information is now linked to this correction notice.

### Supplementary Information

Supplementary Information.

