# Supplementary material for: Author Correction: Visible-light-driven Photocatalytic N-arylation of Imidazole Derivatives and Arylboronic Acids on Cu/graphene catalyst
Source: Sci Rep. 2024 Jan 23;14:2022. doi: 10.1038/s41598-024-51807-z (PMC10805802; doi:10.1038/s41598-024-51807-z)
Supplement: Supplementary file 1 — Supplementary Information. [file 41598_2024_51807_MOESM1_ESM.pdf]

Supporting Information

Visible-light-driven Photocatalytic of N-arylation of Imidazole Derivatives and Arylboronic Acids on Cu/graphene catalyst

Yan-Li Cui, Xiao-Ning Guo*, Ying-Yong Wang, Xiang-Yun Guo*

Figure S1-S8

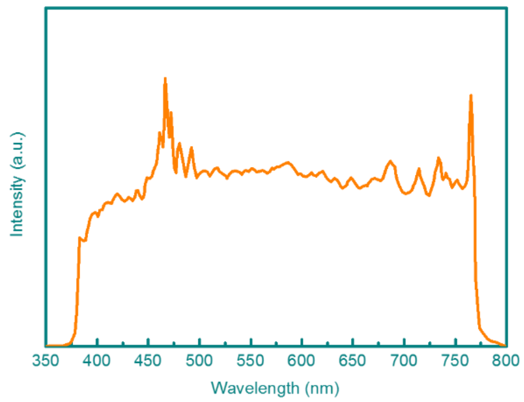

Figure S1 The light spectrum of the Xe lamp, the incident wavelengths mainly range from 400 to 800 nm.

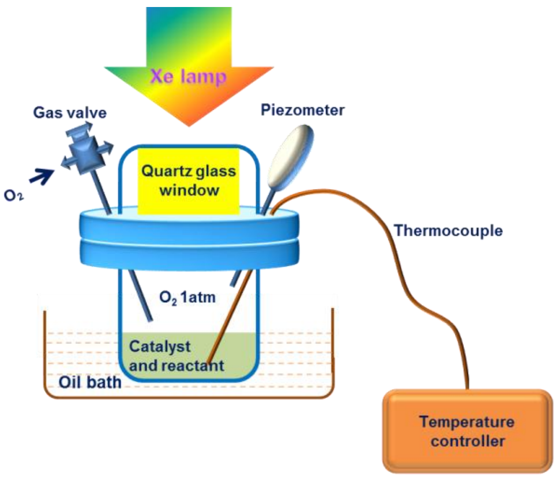

Figure S2 Schematic diagram of photocatalytic N-Arylation of imidazole derivatives and arylboronic acids on Cu/graphene catalyst.

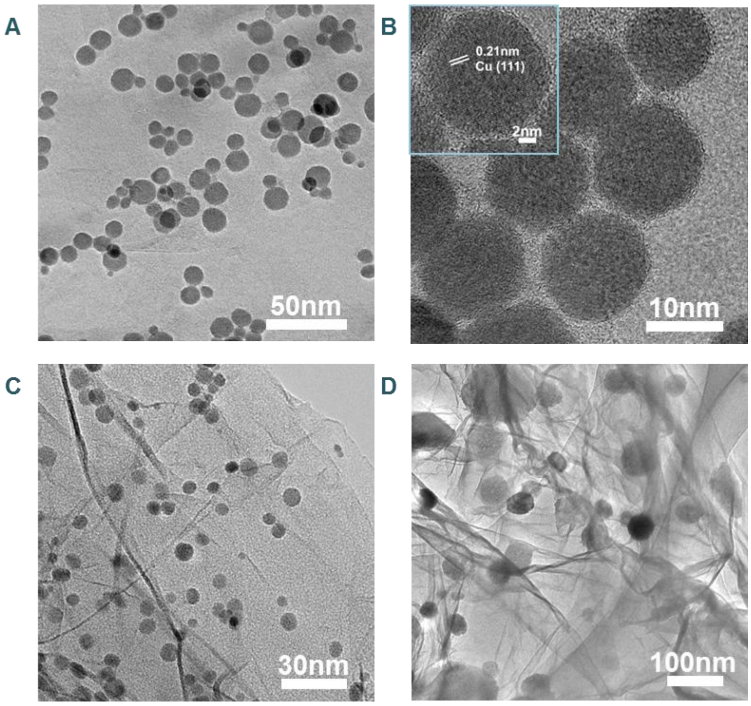

Figure S3 Transmission Electron Microscopy (TEM) images of 5wt% (A and B), 3wt% (C) and 7wt% (D) Cu/graphene catalyst. The insert picture in (B) is a HRTEM image of Cu nanoparticles.

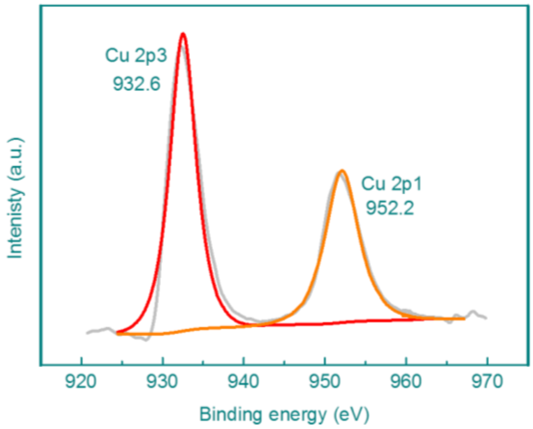

Figure S4 X-ray Photoelectron Spectroscopy profile of the fresh 5wt% Cu/graphene.

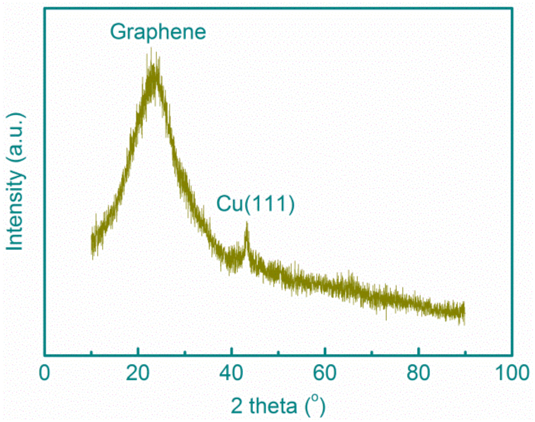

Figure S5 XRD patterns of 5wt% Cu/graphene catalyst confirm that the nanoparticles on graphene sheets are metallic copper.

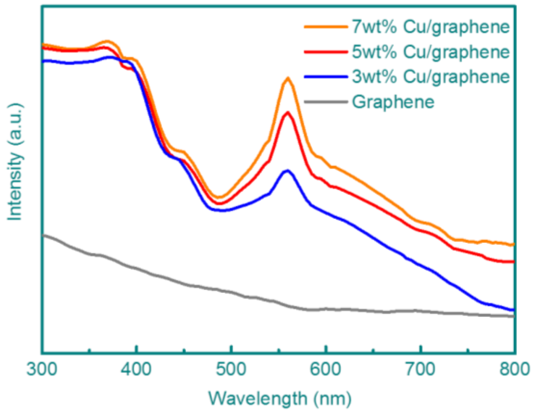

Figure S6 UV-vis absorption spectra of Cu/graphene photocatalysts with different Cu loadings.

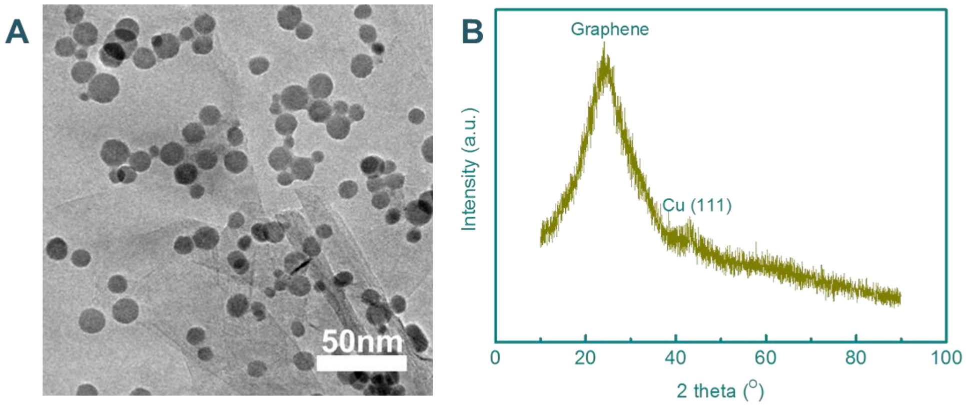

Figure S7 TEM image and XRD patterns of the used 5wt% Cu/graphene after five rounds. The TEM image shows that the Cu nanoparticles are still uniformly dispersed on graphene sheets and their mean size is the same as the fresh one. The XRD results of the used catalyst do not show observable changes in the metallic Cu phase. These results indicate that graphene can effectively stabilize metallic nanoparticles.

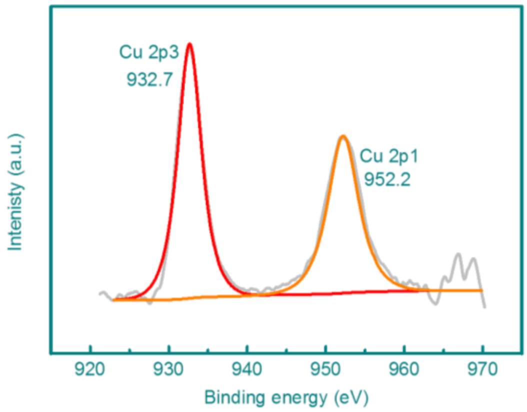

Figure S8 X-ray Photoelectron Spectroscopy profile of the used 5wt% Cu/graphene after five rounds.
